# Prominent and Persistent Extraneural Infection in Human PrP Transgenic Mice Infected with Variant CJD

**DOI:** 10.1371/journal.pone.0001419

**Published:** 2008-01-09

**Authors:** Vincent Béringue, Annick Le Dur, Philippe Tixador, Fabienne Reine, Laurence Lepourry, Armand Perret-Liaudet, Stéphane Haïk, Jean-Luc Vilotte, Michel Fontés, Hubert Laude

**Affiliations:** 1 Institut Scientifique de Recherche Agronomique (INRA), UR892, Virologie Immunologie Moléculaires, Jouy-en-Josas, France; 2 Institut Scientifique de Recherche Agronomique (INRA), UR339, Génétique Biochimique et Cytogénétique, Jouy-en-Josas, France; 3 Service de Neurobiologie, Centre de Biologie et Pathologie Est (CBPE), Groupement Hospitalier Est des Hôpitaux de Lyon, Bron, France; 4 INSERM, Equipe Avenir, Maladies à Prions chez l'Homme, Paris, France; 5 Assistance Publique - Hôpitaux de Paris (AP-HP), Laboratoire de Neuropathologie R. Escourolle, Groupe Hospitalier Pitié- Salpétrière, Paris, France; 6 INSERM UMR 491-IPHM, Faculté de médecine de la Timone, Marseille, France; Columbia University, United States of America

## Abstract

**Background:**

The evolution of the variant Creutzfeldt-Jakob disease (vCJD) epidemic is hazardous to predict due to uncertainty in ascertaining the prevalence of infection and because the disease might remain asymptomatic or produce an alternate, sporadic-like phenotype.

**Methodology/Principal Findings:**

Transgenic mice were produced that overexpress human prion protein with methionine at codon 129, the only allele found so far in vCJD-affected patients. These mice were infected with prions derived from variant and sporadic CJD (sCJD) cases by intracerebral or intraperitoneal route, and transmission efficiency and strain phenotype were analyzed in brain and spleen. We showed that i) the main features of vCJD infection in humans, including a prominent involvement of the lymphoid tissues compared to that in sCJD infection were faithfully reproduced in such mice; ii) transmission of vCJD agent by intracerebral route could lead to the propagation of either vCJD or sCJD-like prion in the brain, whereas vCJD prion was invariably propagated in the spleen, iii) after peripheral exposure, inefficient neuroinvasion was observed, resulting in an asymptomatic infection with life-long persistence of vCJD prion in the spleen at stable and elevated levels.

**Conclusion/Significance:**

Our findings emphasize the possibility that human-to-human transmission of vCJD might produce alternative neuropathogical phenotypes and that lymphoid tissue examination of CJD cases classified as sporadic might reveal an infection by vCJD-type prions. They also provide evidence for the strong propensity of this agent to establish long-lasting, subclinical vCJD infection of lymphoreticular tissues, thus amplifying the risk for iatrogenic transmission.

## Introduction

The variant form of CJD (vCJD), thought to have arisen from exposure of the population to cattle BSE-contaminated food [1], [2], is perceived as the most threatening to public health among the human prion diseases [3]. Nearly two hundred patients have died from vCJD worldwide currently (http://www.cjd.ed.ac.uk/vcjdworld.htm), all of which were homozygous for methionine (M) at the polymorphic *Prnp* codon-129, a genotype harbored by ∼40 percent of the population. The disease incidence has declined since 2000 in the UK, and recent predictions suggest that the epidemic will remain relatively limited in size [4]. However, major uncertainties such as the actual spread of the infectious agent within the population, the proportion of infected individuals who are asymptomatic carriers, and the susceptibility of individuals with non-MM genotypes, render difficult the projections of the future course of vCJD [5]. A retrospective study that aimed to detect abnormal PrP in tonsil and appendix specimens suggested a considerably greater prevalence of vCJD infection than the observed incidence [6], [7]. The pronounced, preclinical involvement of the lymphoid tissues, which appears to be a hallmark of vCJD compared to sporadic CJD (sCJD) infection, conceivably enhances the risk of human-to-human transmission [8]–[11]. Also, BSE/vCJD transmission studies in macaques suggested an increased virulence of the host-adapted agent [12], as generally observed when prions cross a species barrier. For all these reasons and the recent reporting of four vCJD cases associated to blood transfusion [13]–[15], the extend of the iatrogenic risk associated with subclinical vCJD infection needs to be assessed more precisely.

Mice genetically engineered to express human PrP on a nullizygous mouse PrP background have provided new animal models in which to propagate and study the properties of BSE/vCJD agent. Mice that express the PrP allele with M (tg35 and tg45 lines) or V (valine; tg152 line) at codon-129 at supra-physiological levels, while exhibiting a greatly enhanced susceptibility to sCJD compared to wild-type mice, produced a complex pattern of transmission upon BSE or vCJD challenge. Thus, BSE prions were reported to propagate as vCJD-like or sCJD-like prion strains in tg35 mice, while propagation of vCJD in tg152 mice led to the appearance of a distinct strain phenotype [16], [17]. Such findings, together with those from recent molecular strain typing studies [18], raised the concern that propagation of BSE-derived prions in human might result in novel phenotypes. The effect of codon-129 on the susceptibility to human-to-human vCJD transmission was more specifically addressed using a panel of mice in which the mouse PrP gene was replaced by human PrP genes with the MM, VV and MV genotypes [19]. While BSE failed to transmit, vCJD transmitted infection to all three lines though with variable efficiency, strengthening the concern of an iatrogenic risk irrespective of genotype. Despite their potential relevance, such mouse models have remained largely unexploited with respect to the peripheral pathogenesis of BSE/vCJD infection. Indeed, apart from a recent report describing the detection of abnormal PrP in splenic follicular cells of knock-in mice expressing a mouse/human PrP chimera within months after vCJD infection [20], there are no published data regarding the fate of infection after peripheral challenge, or the propagation of prions in extraneural tissues.

In the present study we analyzed the transmission characteristics of vCJD in ‘humanized’ mice, shown to be fully susceptible to the disease. We report the unexpected finding that vCJD prions derived from a natural human isolate can induce the propagation of two distinct prions strains in the brain of mice expressing a homologous PrP protein. We also show that infection in these mice closely parallels vCJD infection in humans with enhanced tropism for lymphoreticular tissues compared to sCJD agent, thus providing a potentially relevant model to investigate the peripheral propagation of vCJD at different stages of the incubation.

## Results

### Efficient disease transmission following intracerebral inoculation of vCJD to tg650 mice

*tg650* is a newly developed transgenic line that overexpresses human PrP^C^ M^129^ allele at a 6-fold level on a PrP null background (see Text S1; Figure S1). To evaluate its susceptibility to vCJD, mice were inoculated intracranially with human infected brain tissue from 3 French cases, and 1 UK case (WHO reference). The resulting transmission data are summarized in table 1, together with those from sCJD cases assayed for comparison. Remarkably, all vCJD inocula induced a clinical neurological disease, with a 100% attack rate. The observed survival times typically averaged 500 days in homozygous mice, and were relatively homogenous overall. Intriguingly however, a proportion of mice inoculated with the case no. 4 developed the disease at a much earlier time (∼300 days) than the others. Such a distribution into two groups (termed *early* and *late* thereafter) was consistently found in homozygous mice, challenged with 2 distinct aliquots of the same material, as well as in hemizygous mice, initially used during the course of the study. Altogether 7 out of the 16 mice infected with vCJD no. 4 developed an early disease (Table 1).

**Table 1:** Transmission of variant and sporadic CJD cases to tg650 mice expressing human PrP.

Inoculum	Number (Origin)	Mean±SEM survival time in days (n/n_0_)^1^		PrP^res^ pattern	
		1^st^ passage	2^nd^ passage	Brain	Spleen
**Variant CJD**	1 (Fr)	522±18 (5/7)^2^	na	vCJD	vCJD
[129 MM]	2 (Fr)	512±15 (8/8)	520±26 (7/7)	vCJD	vCJD
	3 (Fr)	515±41 (8/8)	na	vCJD	vCJD
	4 (UK)^3^	506±21 (6/10)^4^	na	vCJD	vCJD
		338±42 (5/10)^4^	na	sCJD	vCJD
	4 (UK)^5^	628±15 (4/7)^6^	491±37 (7/7)	vCJD	vCJD
		302; 342 (2/7)	154±2 (6/6)	sCJD	vCJD^7^
**Sporadic CJD**	1 (UK)^4^	177±1 (8/8)	153±3 (4/4)	sCJD	neg^8^
[129 MM]	1 (UK)	153±3 (4/4)	157±2 (6/6)	sCJD	neg
	2 (Fr)	159±3 (6/6)	161±2 (6/6)	sCJD	neg
	3 (Fr)	162±3 (6/6)	na	sCJD	neg
	4 (Fr)	167±2 (6/6)	na	sCJD	neg
Human brain^9^	(Fr)	764±35 (0/9)		neg	neg

na, not available; Fr, French; UK, United Kingdom

1 Intracerebral inoculation with 2 mg brain tissue equivalent; n/n_0_: diseased, PrP^res^ positive/inoculated animals.

2 Two tg650 mice died of intercurrent disease at 166 and 274 days but were PrP^vCJD^ positive in both brain and spleen.

3 Done in two independent experiments (different schedule and homogenate batch), of which the results are pooled for the sake of clarity.

4 One tg650 mouse that succumbed with disease at 437 days had a mixed PrP^res^ profile (Figure S4) and was included for calculation of both survival times.

5 1^st^ passage performed on hemizygous mice.

6 One tg650 mouse died of intercurrent disease at 411 days, but was PrP^vCJD^ positive both in brain and spleen.

7 All mice and half of the mice accumulated vCJD-like PrP^res^ in their spleen on 1^st^ and 2^nd^ passage, respectively. The others were negative.

8 neg: negative.

9 Patient with no TSE disease.

To clarify the situation observed in vCJD no. 4-challenged mice, *late* and *early* brain materials from individual mice (sacrificed at 586 and 302 days, respectively) were subjected to a secondary transmission (see Table 1). *Late* brain-inoculated mice died at ∼500 days, a survival time that neither changed on 3^rd^ passage (497±18; 10/10 mice) nor differed significantly from that seen upon primary transmission of vCJD on homozygous mice (Table 1). A much shorter incubation period (∼160 days), similar to that with secondary passed sCJD was observed upon *early* brain passage (Table 1), suggesting that distinct prions had propagated in *early* and *late* mice.

### PrP^res^ in *early* succumbing *tg650* mice is sCJD-type in the brain but vCJD-type in the spleen

**Figure 1:**
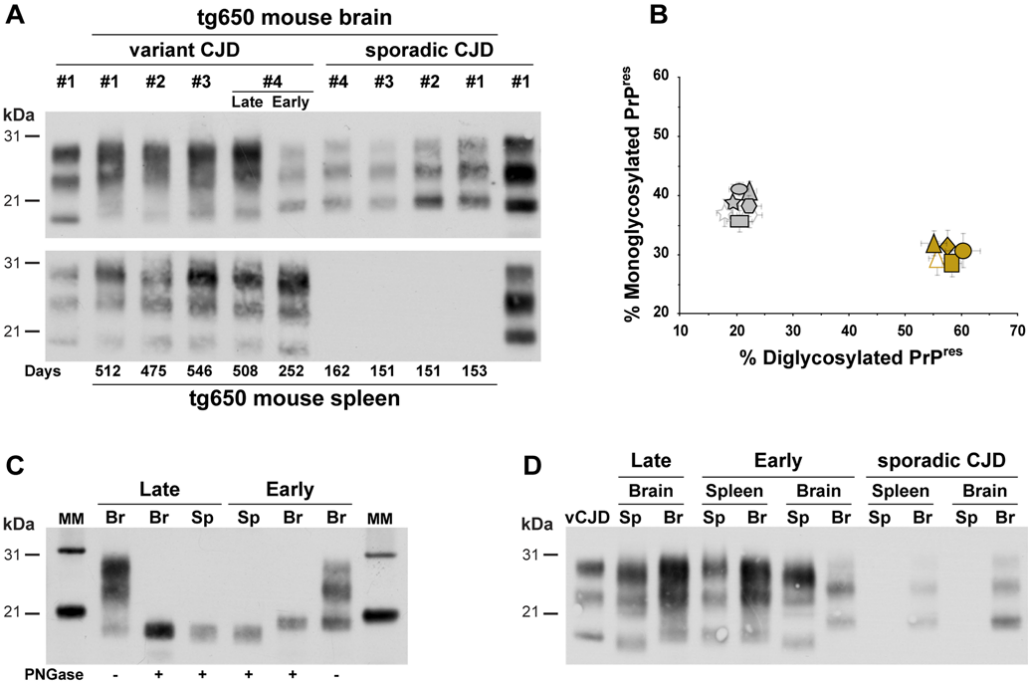
Western blots analysis of PrP^res^ in the brains and spleens of *tg650* mice infected with sporadic and variant CJD by intracerebral route. (A) Electrophoretic pattern of PrP^res^ in the brain (upper panel) and spleen (lower panel) upon primary transmission. Note that vCJD case no. 4 shows either a typical vCJD or sCJD-like pattern in the brains of mice succumbing late or early with disease, respectively, while a vCJD-like pattern is consistently observed in the spleens. The PrP^res^ profiles of variant and sporadic CJD in the brain of affected human patients are shown on the sides (left and right, respectively) of both immunoblots for comparison. (B) Ratio of diglycosylated and monoglycosylated PrP^res^ species in the brains of mice following serial transmission of sporadic or variant CJD cases (data plotted as means±SEM). PrP^vCJD^ and PrP^sCJD^ patterns are represented by brown and grey colors, respectively. Variant CJD cases no. 1, 2, 3 and 4 are represented as diamond, circle, square, and triangle; sCJD cases no. 1, 2, 3 and 4 as polygon, star, ellipse, and rectangle. Secondary transmissions (when available) are represented by the same, unfilled symbols. Note the distinct glycoform ratio between *early* (grey triangle) and *late* brains upon transmission of vCJD case no. 4. (C) In *early* succumbing mice, the size of the PrP^res^ fragments is higher in the brain (Br) than in the spleen (Sp), whereas in *late* succumbing it remains similar, as shown after deglycosylation by PNGase treatment. MM: molecular markers. (D) Electrophoretic pattern of PrP^res^ in the brain and spleen upon secondary transmission. *tg650* mice were inoculated with brain or spleen homogenates from either sCJD or vCJD case 4 *late* or *early* mice. The brain PrP^res^ profile of human vCJD is shown on the left side of the gel for comparison.

The nervous and lymphoid tissues were examined for the presence of proteinase K (PK)-resistant PrP (PrP^res^) by immunoblotting. PrP^res^ was readily detected in the brains of all the vCJD- and sCJD-infected mice analyzed. A typical PrP^vCJD^ banding pattern, characterized by low size fragments and prominent diglycoform species, was observed in vCJD no. 1 to 3-challenged mice and in vCJD no. 4 *late* mice (23/23 and 9/9 mouse brains analyzed, respectively, Figure 1A–B), a signature that was maintained in the brain of secondary inoculated mice (6/6 analyzed; Figure 1B, D). vCJD no. 4 *early* mice, however, showed a clearly distinct PrP^res^ profile with predominantly monoglycosylated, higher size fragments (6/6; Figure 1A–C). Such a signature, termed PrP^sCJD^ because of its similarity with that in sCJD-challenged mice was conserved on subsequent passage (35/35 analyzed; Figure 1A, B, D).

While no PrP^res^ accumulation was evident in the spleens of sCJD-infected mice (12/12 analyzed), PrP^res^ was consistently detected in the spleens from vCJD-infected mice (32/32) (Figure 1A; Table 1). Remarkably, *early* and *late* mice showed a similar PrP^res^ signature, unlike that in the corresponding brains (Figure 1A–C). The observed banding pattern was typical of PrP^vCJD^, despite of a slightly modified glycoform ratio compared to that in the brain, as already noted in human infected patients [10]. PrP^vCJD^ was also detected in the spleens of half of the mice (2/4) inoculated with *early* mouse brain (Table 1 and Figure 1D), thus suggesting that vCJD type agent was replicated as a minor component in the brains of a number of *early* mice and then preferentially amplified in the spleen on subpassage.

### vCJD propagated as sporadic- or variant-like CJD strain in the brain of tg650 mice

**Figure 2:**
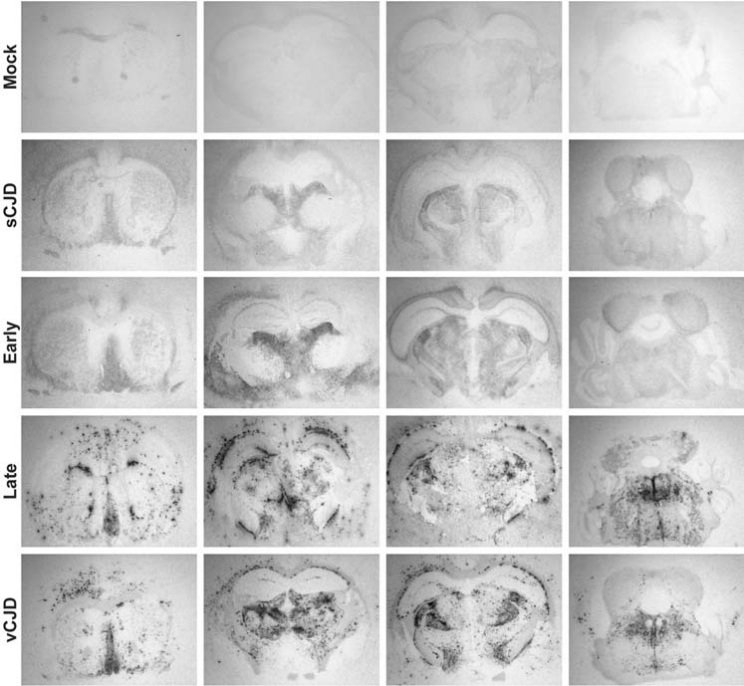
Regional distribution of PrP^res^ in the brains of *tg650* mice infected with vCJD and sCJD. The distribution of PrP^res^ deposits in the brains of tg650 mice infected with vCJD no. 4 *late* or *early* brain, vCJD (case no. 1), and sCJD (case no. 2) or non-TSE material (mock) is shown on representative histoblots in 4 different antero-posterior sections. Note the differing size and distribution of PrP^res^ deposits between *late* and *early* brain, and their similarity to that observed with vCJD and sCJD, respectively.

To confirm that the biological and molecular disparities observed after transmission of vCJD no. 4 reflected the propagation of two distinct prion strains, we examined the regional distribution of abnormal PrP and spongiform lesions in the brain, which are known to exhibit strain-dependant variations. We first performed histoblots analyses on antero-posterior coronal frozen sections to compare the distribution of PrP^res^ in the brains of mice inoculated with *late* or *early* brain. In *late* brain passage, abundant and large, plaque-like PrP deposits were scattered throughout the brain, predominantly in the cerebral cortex, corpus callosum, several nuclei of the thalamus, optic tract and brain stem, a distribution similar to that seen in vCJD 1–3 brains (Figure 2; Table S1). In sharp contrast, PrP deposition in *early* brain passage was absent in most of these regions (Figure 2; Table S1). The staining was much less intense, rather diffuse, but nevertheless specific as compared to mock-infected mice (Figure 2). It was restricted to the ventral pallidum, dorsolateral geniculate, lateral posterior and mediodorsal nuclei of the thalamus and external cortex of the inferior colliculus, resembling that in sCJD 1–3 brains (Figure 2; Table S1).

**Figure 3:**
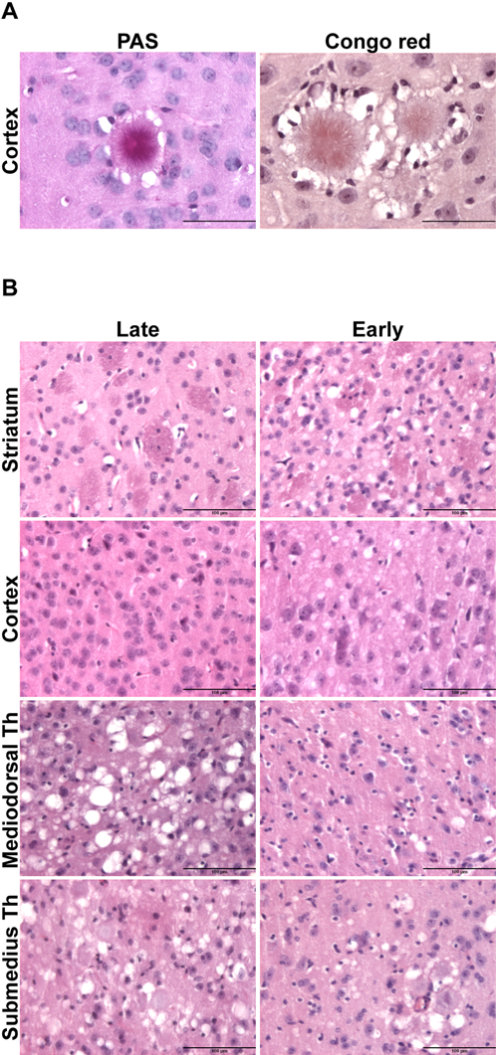
PrP^res^ plaques and spongiform changes in mice infected with vCJD no. 4 *late* or *early* brain. (A) PrP plaques were visible, here in the cortex of *late* brain passage, after periodic acid-Schiff (PAS) and Congo red staining. Note that their morphology and the surrounding ring of vacuoles are typical of florid plaques, a pathological hallmark of vCJD in human. (B) Hematoxylin and eosin staining of brain sections from mice infected with *late* or *early* brain. Note the distinct regionalization of vacuolation among the two agents. Th; thalamus. Bars: (A) = 200 µm. (B) = 100 µm.

The distinct nature of abnormal PrP deposits was further assessed by light and fluorescent microscopy examination of fixed and frozen sections. Numerous plaques were present only in *late* brain passage (Figure S2). Their structure had a typical aspect arranged in peripheral radiating spicules (Figures 3A and S3). They were Congo red (Figure 3A) and thioflavin-S positive (Figure S3), indicating an amyloid fibrils organization. A large number of these dense deposits were surrounded by a ring of vacuoles (Figure 3A), a morphology typical of the florid plaques observed in vCJD-infected humans [1]. Examination of histopathologic lesions in several areas of the brain indicated that spongiosis was prominent in the thalamus of *late* brain passage, whereas striatum and cortex were mostly damaged in *early* brain passage (Figure 3B). Astrogliosis activation was important in the thalamus and cortex frequently at the vicinity of the plaques in *late* brain, whereas it was spatially unrelated to PrP^res^ deposition in *early* mice (Figure S2). In one of the *early* brains, the observed PrP distribution suggested the presence of both PrP^sCJD^ and PrP^vCJD^, consistent with the mixed PrP^res^ electrophoretic pattern visible on the immunoblot (Figure S4).

### Long-lasting persistence of vCJD prion in the spleen following peripheral inoculation

**Figure 4:**
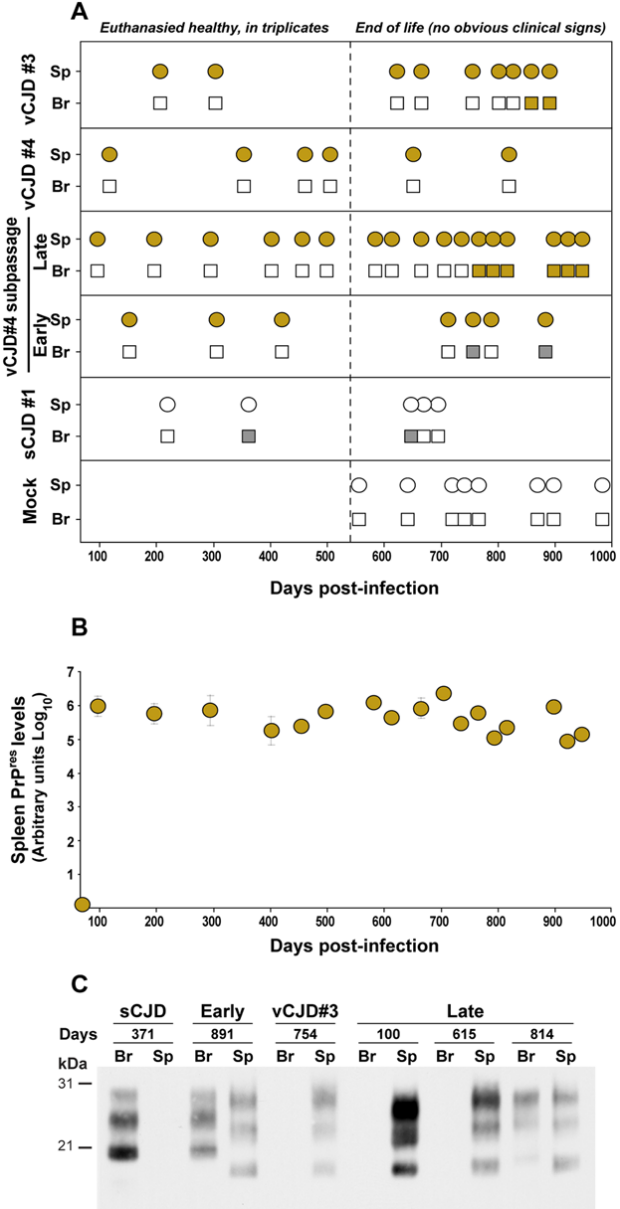
PrP^res^ accumulation in *tg650* mice intraperitoneally infected with vCJD. (A) PrP^res^ electrophoretic pattern in spleen (sp, circle) and brain (br, square) of mice euthanised healthy in triplicates up to 550 days post-infection (broken line) or at end of life time. vCJD- and sCJD-associated PrP^res^ pattern are represented as brown and grey color, respectively. Empty symbols indicate PrP^res^ negative tissue. Mock-infected mice (mock) were intraperitoneally inoculated with human brain without any TSE. (B) Quantification of PrP^res^ levels in the spleens on secondary transmission of vCJD (*late* brain). Spleen tissue was collected from healthy mice euthanised in triplicates at regular time points or individually at the end of life (see above). PrP^res^ was extracted, detected by western blot and quantified with a bio-imaging system for chemiluminescence applications (see Methods). Results are expressed in arbitrary units (logarithmic scale). The standard deviations for triplicates without error bars were too small to be represented. (C) Electrophoretic pattern of PrP^res^ in brain (Br) spleen (Sp) at various times after inoculation of the indicated brain materials.

We next sought to investigate the pathogenesis of the infection following inoculation of tg650 mice by the peripheral route. Mice were inoculated intraperitoneally with brain material from vCJD- or sCJD-infected humans and from vCJD no. 4 mouse passages. Animals were euthanised at various times post-infection (pi), from which the brain and spleen were collected for PrP^res^ analysis by immunoblotting. Importantly, mice inoculated with vCJD prions peripherally appeared not to develop clinical disease, unlike their intracranially inoculated counterparts. Yet PrP^vCJD^ was readily detected in the spleen, and this from 100 days pi onward (Figure 4A). Moreover, quantitative analysis revealed that the PrP^res^ levels did not greatly vary during the life of the mouse (Figure 4B–C), except perhaps a slight decrease at late stage of infection, possibly due to age-related impairment of the follicular dendritic cells (for review [21]) that support PrP^res^ accumulation in spleen [22]. In the brain, PrP^res^ was detected only lately and in a proportion of the mice; its banding pattern was of vCJD type in *late* mice and of sCJD type in *early* mice subpassage, like following intracerebral inoculation (Figure 4A, C). In contrast, even after intraperitoneal challenge, we failed to detect PrP^res^ accumulation in the spleens of sCJD-infected mice (Figure 4C).

In order to evaluate the level of prion infectivity present in this compartment, spleen homogenates of mice challenged intracerebrally with either sCJD or vCJD cases were subpassaged intracranially to recipient tg650 mice (at 10% w/v). The mice challenged with sCJD spleens died at ∼300 days (case no. 1: 296±8 days, 5/6 mice infected; case no. 4: 334±6 days, 6/6), corresponding to infectivity levels ∼10^4^-fold lower than in the brain by applying these values to a dose/survival curve drawn after an endpoint titration (Figure S5). These low levels were consistent with the lack of detectable PrP^res^ (Figures 1 and 4) In sharp contrast, the mice challenged with vCJD spleen died at 500±61 days (6/6), a survival time intermediate between that observed after inoculation of vCJD brain at the 10% and 1% doses (497±18; (10/10 mice) and 537±13 days; (7/7), respectively). The PrP^res^ profiles in the brains and spleens of the reporter mice were consistent with the replication of sCJD and vCJD agents, respectively (Figure 1D). Thus vCJD infectivity levels in the spleen and brain of tg650 mice are unlikely to differ by more than about 1 order of magnitude. Such an estimate is consistent with the PrP^res^ level ratios in spleen versus brain homogenates (between 5 to 20-fold less). It is notable that this PrP^res^ ratio is close from that reported in humans [10]. Moreover, inoculation of vCJD-infected human spleen tissue killed tg650 mice within 570±63 days (5/5), which indicates an infectivity level approaching that in tg650 mouse spleen. In contrast, infectivity levels 100- to 1000-fold lower than in the brain were reported for vCJD-infected human spleen tissue through titration in RIII mice [23].

## Discussion

In this study we used tg650 mice, a newly developed transgenic line expressing human PrP^C^, to investigate some aspects of the pathogenesis of vCJD infection. As main findings, we demonstrate that prion strain divergence can occur upon transmission of human, primary vCJD to such mice, and that peripheral challenge leads to an asymptomatic, life-long infection of the lymphoid compartment. A feature of tg650 mice is that following primary intracerebral vCJD challenge they developed a neurological disease with typically 100% attack rate, unlike for previously established PrP^129Met^, including overexpressing lines [16], [19]. The mean survival time – typically around 500 days in homozygous mice - did not change notably on subpassaging, implying that vCJD agent might clinically infect the tg650 mice with little or no transmission barrier. This discrepant result may reflect the use of different constructs and genetic backgrounds (Text S1), and the transgene expression levels, although the latter does not seem to greatly differ as far as the tg650^+/−^ and tg45 mice [16] are concerned.

A surprising result of these studies is the alternate pattern of disease that was induced by one of the inoculated vCJD cases, a WHO reference case here designated vCJD no. 4. Indeed, while vCJD strain features were faithfully propagated in the majority of tg650 mice, almost half of the vCJD 4-inoculated mice were found to propagate a prion replicating faster than vCJD agent, and exhibiting sCJD-like PrP^res^ and neuropathological features. Although strain divergence upon transmission of BSE/vCJD agent to mice was reported to occur in earlier studies [16], [24], it was unprecedented within a context of homotypic transmission, i.e. full matching between the donor and receiver PrP sequences. To address the issue of a possible contamination, we performed independent transmission experiments, involving separate inoculum batches of the incriminated case, which all produced consistent results. Therefore, we consider the data inconsistent with contamination of the VCJD no. 4 material by a sCJD infectious source within our laboratory. An alternate possibility, i.e. a cross-contamination of the source material, was judged highly improbable owing to the procedures applied during the collect of the specimen and the preparation of the homogenates ([25] and P. Minor, personal communication). On the other hand, our observation intriguingly parallels the phenotypic disjunction observed upon transmission of BSE agent to human PrP^129Met^ mice (tg35 line [16]). Together, these findings lend support to the hypothesis that a minor strain component might be created upon cattle-to-human transmission of BSE agent and could emerge upon subsequent human-to–human transmission. It is also worth mentioning that, while the probability to detect such a variant through mouse bioassay would be expected to depend on the amount - and possibly the regions - of brain tissue taken to establish the source material, the vCJD-4 homogenate was prepared using a larger amount of tissue from the same brain than for the other homogenates analyzed in this study (i.e. 100 mg instead of 1 mg of frontal cortex [25]).

The above finding has obvious implications in terms of public health as it raises the concern that some humans iatrogenically infected by vCJD agent may develop a clinical disease that would not be recognized as of vCJD origin [17], [26]. Strikingly however, all vCJD-4-inoculated mice, notwithstanding the strain phenotype divergence propagated *bona fide* vCJD agent in their spleen, based on the PrP^res^ pattern and the disease phenotype produced by secondary transmission to tg650 mice. This result is of direct relevance to the diagnosis of variant and sporadic CJD. Indeed, looking for peripheral lymphoreticular deposition of abnormal PrP on cases diagnosed as sporadic CJD might reveal a vCJD infection resulting from human-to-human, or cattle-to-human transmission. In this respect, it would be of interest to examine whether BSE-inoculated tg35 mice showing discordant PrP^res^ signatures [16], or vCJD-challenged PrP^129Val^ transgenic mice producing ‘type 5’ prion in their brain [17] do accumulate PrP^vCJD^ in their spleens. In any case, our findings provide clear evidence that, as a consequence of strain-related tropism disparities, the same mouse can propagate different prions in different tissues following a single infection event.

Another salient finding emerging from this study was the remarkable ability of vCJD agent to establish asymptomatic infection despite sustained, life-long propagation in extraneural tissues. When challenged peripherally, tg650 mice remained asymptomatic over the whole observation period, and did not accumulate PrP^res^ at detectable levels in their brain before 750 days pi, near the life end-stage. In the spleen of these mice however, PrP^res^ accumulation reached its maximum at an early stage of infection, and remained at stable and substantial levels until death. Plateauing of prion infection in the spleen is consistent with earlier observations, and has been suggested to reflect an exhaustion of target cells (for review [22]) Importantly, the spleen tissue was highly infectious as it killed 100% of intracerebrally challenged mice within the minimal mean incubation time (∼500 days). Altogether these data support the view that the sustained multiplication of the vCJD prion in lymphoid tissues was not accompanied by an efficient neuroinvasion in tg650 mice. Such an extremely delayed neuroinvasion appears to be rare in TSE rodent models, and to our knowledge was only reported for the mouse-adapted strain 87V on IM mice infected intraperitoneally with diluted inoculum [27]. Clearly, while early accumulation of prions in lymphoid tissues may be essential for efficient neuroinvasion [22], efficient lymphoinvasion does not inevitably lead to rapid neuroinvasion. This finding strengthens the notion that humans infected by vCJD from a human source – including individuals of the MM genotype – might remain clinically asymptomatic for a very prolonged period of time while harboring relatively high levels of prion infectivity in their lymphoid tissues from an early stage of infection on, thereby amplifying the risk of iatrogenic transmission. It also supports the view that the large-scale survey of lymphoreticular tissues [28] may lead to a reliable assessment of the actual prevalence of vCJD infection in the UK population.

Finally, the human PrP transgenic model described in this study may help to further our understanding of peripheral vCJD pathogenesis, for instance in trying to identify factors that might enhance neuroinvasion efficiency, or modulate the shedding of prion infectivity from the lymphoreticular to the blood compartment. Moreover, preliminary results indicate that the search for abnormal PrP in the spleen of such mice culled at time intervals post infection [29], [30] could allow the detection of low levels of vCJD infectivity within a reasonably short time scale.

## Methods

### CJD samples

Two WHO reference brain samples sCJD no. 1 (code NHBY0/0001) and vCJD no. 4 (code NHBY0/0003) [25] and a vCJD-patient spleen (code NHSY0/0009) were provided by the National Institute for Biological Standards and Control (NIBSC; Potters Bar, United Kingdom). The other samples were collected in French hospitals. All samples consisted of frontal cortex from patients homozygous for methionine at PrP codon-129. Consent for use of these materials for research was obtained.

### Mouse transmission assays

Details of how the tg650 transgenic mouse line was created are supplied as supplementary information (Text S1). Animal experiments have been performed according to our institutional and national guidelines. Rigorous protocols were followed throughout the study to avoid any cross-contamination in the laboratory. Each inoculum was prepared extemporaneously in a class II microbiological cabinet using disposable equipment. 20 µl of a 10% or 100 µl of a 2% (wt/vol) brain homogenate in 5% glucose were inoculated in individually identified 6- to 10-week old mice by intracerebral or intraperitoneal route, respectively, using disposable syringes. Mice were sequentially euthanised to study the kinetics of PrP^res^ accumulation in brain and spleen after intraperitoneal injection. Otherwise mice were monitored daily and those showing disease signs or at end of lifespan were euthanised *in extremis*. Separate instruments were used for brain and spleen tissues. Instruments were decontaminated with 1M sodium hydroxide for 24 h followed by double autoclaving at 136°C before re-use. All collected tissues were identified individually by a database code with highly resistant thermal labels designed for long-term storage (Brady Corp).

### PrP^res^ immunoblots

All procedures regarding extraction and detection of PrP^res^ from brains and spleens of infected mice have been previously described [31], [32]. The ICSM18 [33] or Sha31 [34] anti-PrP antibodies were used. Enzymatic deglycosylation was performed on denatured PrP^res^ with 1,000 U of recombinant PNGase (New England Biolabs) for 2 h at 37°C in 1% Nonidet P40 and the proprietary buffer as described [33]. Glycoform ratios, apparent molecular mass and quantification of PrP^res^ signals were determined by the GeneTools software after acquisition of chemiluminescent signals with a GeneGnome digital imager (Syngene).

### Histoblots

Brains were rapidly removed from euthanised mice and frozen on dry ice. Thick 8–10 µm cryostat sections were cut, transferred onto Superfrost slides and kept at −20°C until use. Histoblot analyses were performed on 3 brains per infection, using the 3F4 anti-PrP antibody as described [31].

### Immunofluorescence

Cryostat sections were formalin-fixed before permeabilisation with 0.5% Triton X-100 for an hour. Abnormal PrP epitopes were exposed with 3M guanidine-HCl for 5 min [35]. Endogenous avidin and biotin were blocked using a specific kit (Zymed). PrP^res^ was detected with ∼2 µg/ml biotinylated 3F4. Astrocyte activation was visualized by labeling with anti-glial fibrillary acidic protein (GFAP, Dako). Sections were incubated with appropriate Alexa-conjugated streptavidin or secondary antibodies (Molecular probes) and nuclear marker 4′, 6-diamidino-2-phenylindole (Sigma) before mounting in fluoromount (Sigma). Age-matched uninfected tg650 mice were similarly stained as controls. Signals were acquired with an inverted fluorescence microscope (Zeiss Axiovert) and analyzed with the Metamorph software.

### Histopathology

Brains were removed from euthanised mice and the 2/3 were fixed in 4% phosphate buffered formalin. Seven-micrometer–thick sections from paraffin embedded samples were stained with hematoxylin and eosin, periodic acid-Schiff and Congo red [36]. Thioflavin-S staining (Sigma) was performed as previously described [37], [38] with slight modifications of method. Briefly, after a pre-treatment by immersion in 0.1% KMnO_4_ in PBS for 20 min followed by 1% oxalic acid in PBS for 3 min and 1% H_2_O_2_ and NaOH in PBS for 5 min, sections were stained using a 0.01% solution of Thioflavin-S in 40% EtOH. Finally sections were differentiated in 50% EtOH in PBS, rinsed in PBS, coversplipped and observed using a Zeiss Axiovert 200M microscope equipped with a Coolsnap HQ CDD camera and using Metaview software.

## Supporting Information

Figure S1 Relative PrPC levels in the brain and spleen of tg650 and C57BL/6 mice
(0.27 MB PDF)

Figure S2 Immunofluorescent labeling of frozen coronal sections of brains from mice infected with late or early mouse brain or non-TSE material
(6.25 MB PDF)

Figure S3 Periodic acid-Schiff and thioflavin-S staining of fixed coronal brain sections of mice infected with late brain
(7.95 MB PDF)

Figure S4 Analysis of a mouse infected with vCJD case no. 4 and showing a mixed PrPres profile
(2.63 MB PDF)

Figure S5 End point titration of sCJD infectivity in tg650 mice
(0.27 MB PDF)

Table S1 Differential distribution of PrPres in the brains of tg650 mice infected with early or late brain passage
(0.05 MB DOC)

Text S1 Development of the human PrP transgenic mice (tg650 line)
(0.03 MB DOC)
