# Supplementary material for: Prominent and Persistent Extraneural Infection in Human PrP Transgenic Mice Infected with Variant CJD
Source: PLoS One. 2008 Jan 9;3(1):e1419. doi: 10.1371/journal.pone.0001419 (PMC2171367; doi:10.1371/journal.pone.0001419)
Supplement: Figure S1 — Relative PrPC levels in the brain and spleen of tg650 and C57BL/6 mice (0.27 MB PDF) [file pone.0001419.s001.pdf]

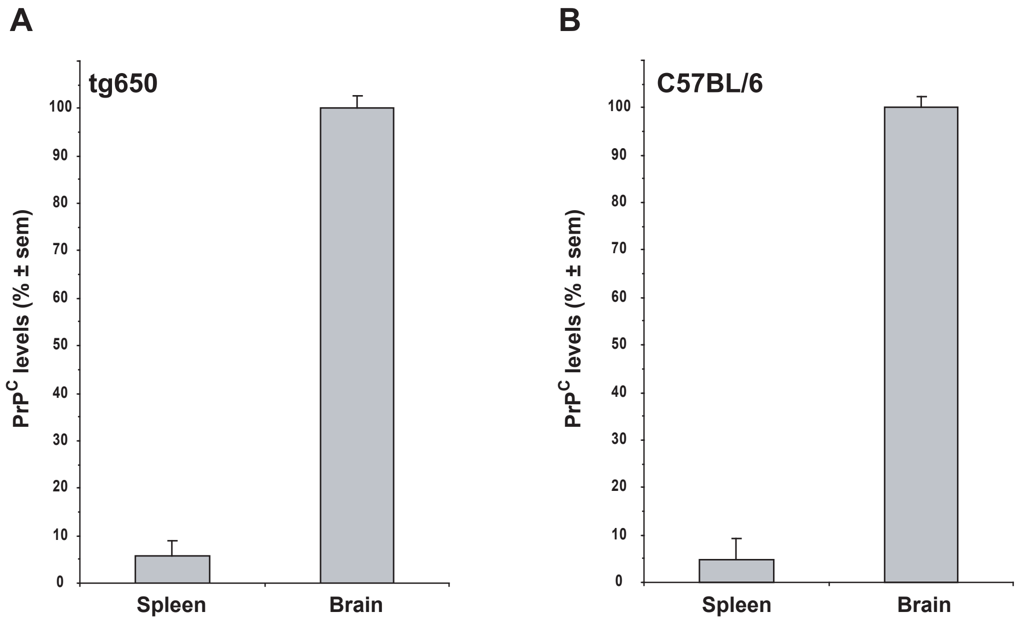

Figure S1. Relative PrP^C levels in the brain and spleen of tg650 (A) and C57BL/6 mice (B).

Quantification was performed by western blot on 3 mice. The results are expressed as mean ± SEM.
