# Supplementary material for: Prominent and Persistent Extraneural Infection in Human PrP Transgenic Mice Infected with Variant CJD
Source: PLoS One. 2008 Jan 9;3(1):e1419. doi: 10.1371/journal.pone.0001419 (PMC2171367; doi:10.1371/journal.pone.0001419)
Supplement: Figure S2 — Immunofluorescent labeling of frozen coronal sections of brains from mice infected with late or early mouse brain or non-TSE material (6.25 MB PDF) [file pone.0001419.s002.pdf]

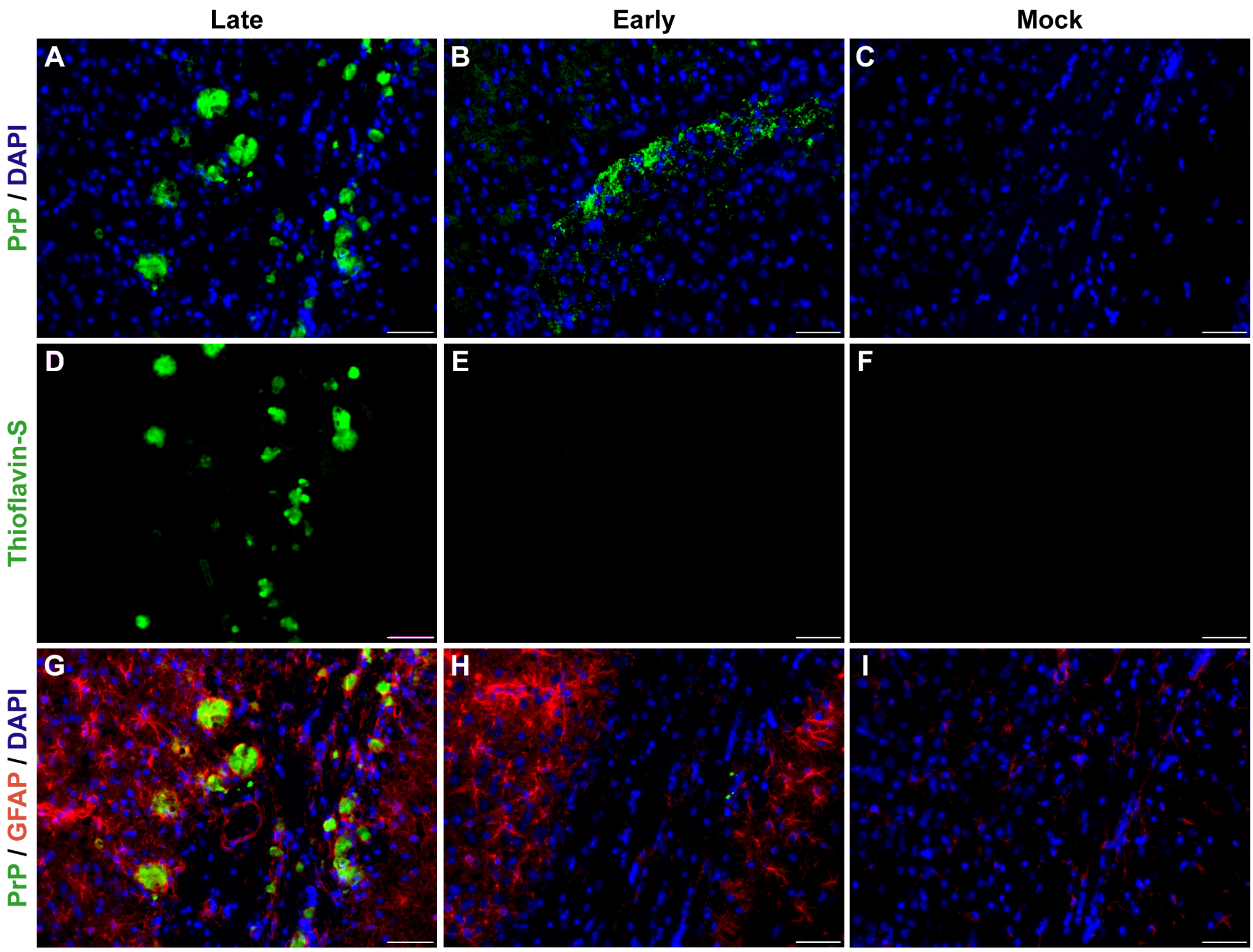

Figure S2. Immunofluorescent labeling of frozen coronal sections of brains from mice infected with *late* or *early* mouse brain or non-TSE material (mock).

(A-C) PrP^{Sc} (Alexa 488, green) and nuclear marker 4',6-diamidino-2-phenylindole (DAPI, blue) staining at the level of the corpus callosum (A, C) or the dorsal lateral geniculate nucleus of the thalamus (B).

(D-F) Thioflavin-S staining (green) of sections immediately adjacent to (A-C). Note the amyloid (D) nature of PrP^{Sc} deposits in the brain of mice infected with *late* brain.

(G-I) PrP^{Sc} (Alexa 488, green), GFAP (Alexa 568, red) and nuclear marker DAPI (blue) staining at the level of the corpus callosum (E, F). Note the pronounced astrogliosis in terminally ill mice (G, H), independently of PrP^{Sc} accumulation in those infected with *early* brain (H). Scale bar = 50 µm.
