# Supplementary material for: Prominent and Persistent Extraneural Infection in Human PrP Transgenic Mice Infected with Variant CJD
Source: PLoS One. 2008 Jan 9;3(1):e1419. doi: 10.1371/journal.pone.0001419 (PMC2171367; doi:10.1371/journal.pone.0001419)
Supplement: Figure S3 — Periodic acid-Schiff and thioflavin-S staining of fixed coronal brain sections of mice infected with late brain (7.95 MB PDF) [file pone.0001419.s003.pdf]

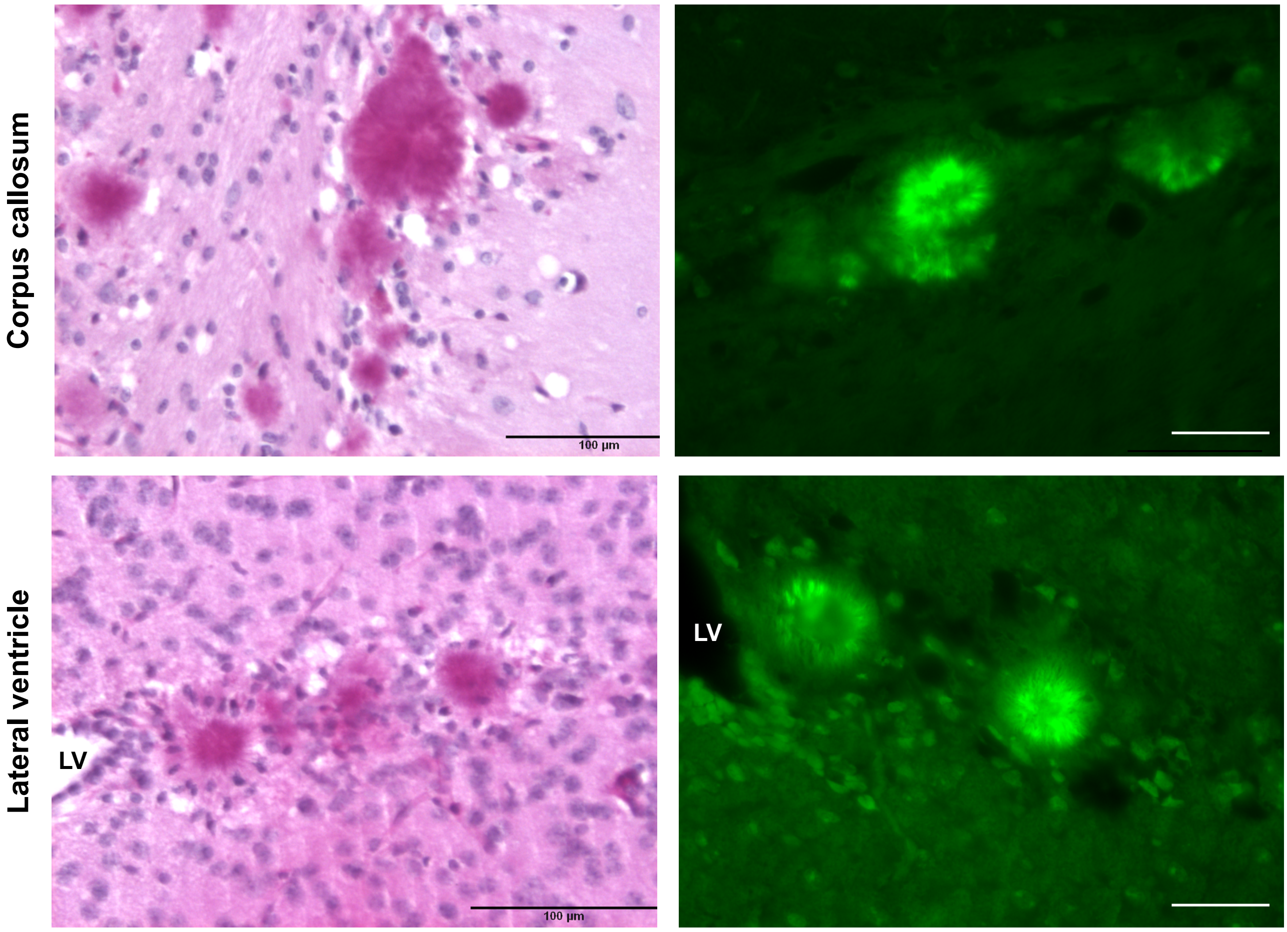

Figure S3. Periodic acid-Schiff (left panel) and thioflavin-S staining (right panel) of fixed coronal brain sections of mice infected with *late* brain.

Sections were stained at the level of the corpus callosum or the lateral ventricle (LV). Note the typical aspect of the plaques, arranged in peripheral radiating spicules. Scale bars = 100 µm.
