# Supplementary material for: Prominent and Persistent Extraneural Infection in Human PrP Transgenic Mice Infected with Variant CJD
Source: PLoS One. 2008 Jan 9;3(1):e1419. doi: 10.1371/journal.pone.0001419 (PMC2171367; doi:10.1371/journal.pone.0001419)
Supplement: Figure S4 — Analysis of a mouse infected with vCJD case no. 4 and showing a mixed PrPres profile (2.63 MB PDF) [file pone.0001419.s004.pdf]

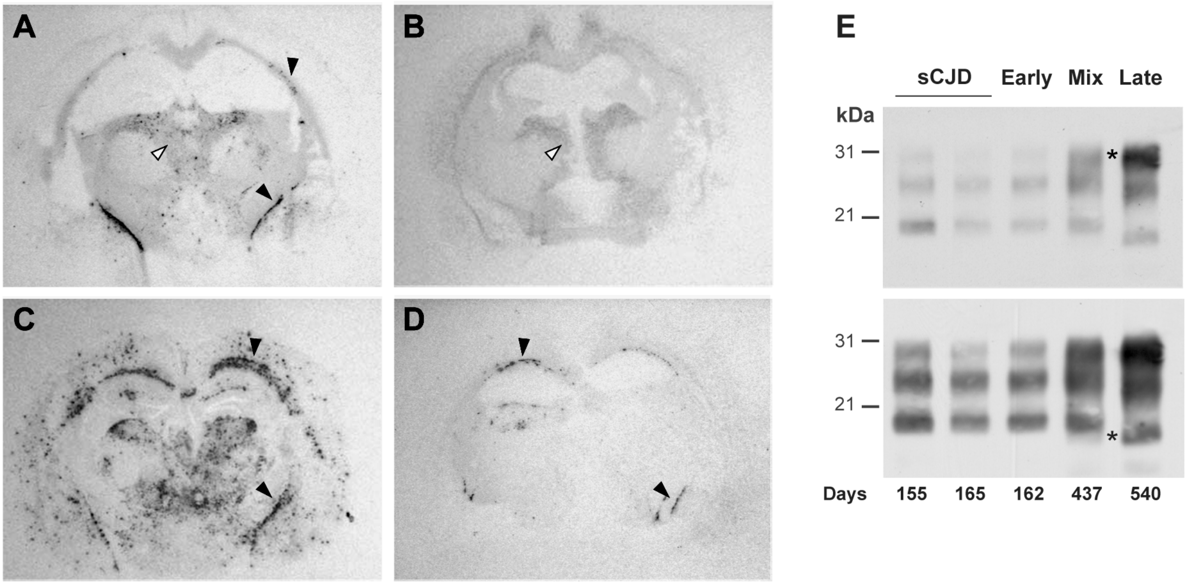

Figure S4. Analysis of a mouse infected with vCJD case no.4 and showing a mixed PrP^{res} profile.

(A) Histoblot of brain sections of a diseased mouse (437 days pi), showing both diffuse (white arrowheads) and granular (black arrowheads) PrP^{res} deposits. The distribution suggests a mixture of PrP^{sCJD} and PrP^{vCJD} when compared to mice infected with sCJD (B) or with vCJD (C: terminal stage; D: culled at 300 days pi).

The corresponding immunoblot is shown in E (lane 4, 'mix'), in comparison to sCJD, early and late brains. Note the enrichment in diglycosylated PrP^{res} species and the apparition of a faster migrating unglycosylated band (stars) on a more exposed gel bottom. PrP^{res} was extracted from brain material harvested after slicing of the sections used for histoblotting.
