# Supplementary material for: Prominent and Persistent Extraneural Infection in Human PrP Transgenic Mice Infected with Variant CJD
Source: PLoS One. 2008 Jan 9;3(1):e1419. doi: 10.1371/journal.pone.0001419 (PMC2171367; doi:10.1371/journal.pone.0001419)
Supplement: Figure S5 — End point titration of sCJD infectivity in tg650 mice (0.27 MB PDF) [file pone.0001419.s005.pdf]

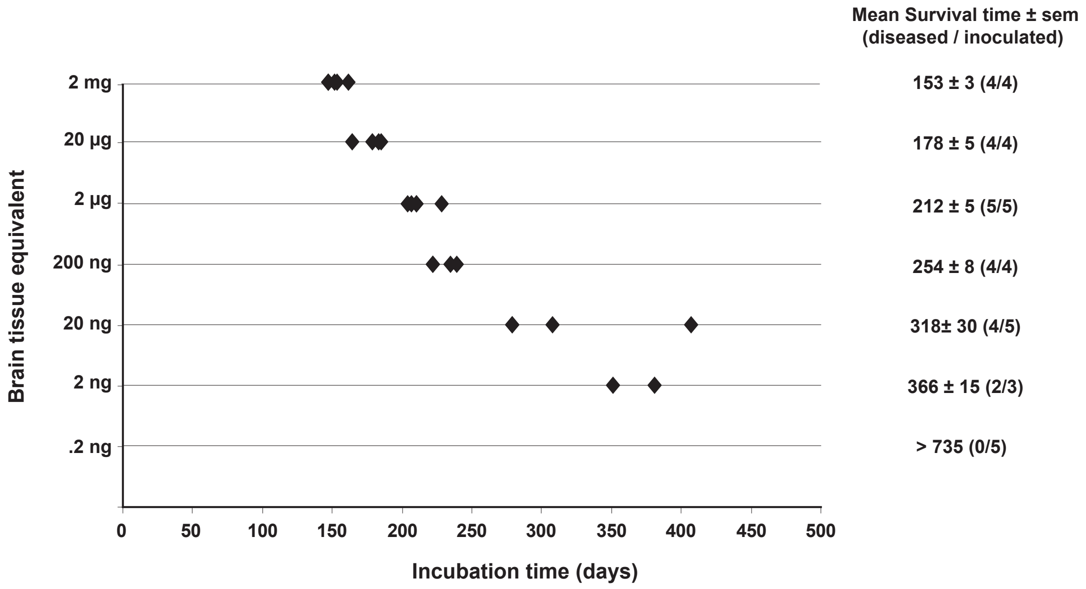


**Figure S5. End point titration of sCJD infectivity in tg650 mice.**

Brain homogenate from tg650 mice infected with sCJD brain was serially diluted and inoculated intracerebrally. The corresponding amounts of brain equivalent material, the mean survival time (days ± sem) and the number of diseased/inoculated mice are indicated. The diseased mice were positive for brain $PrP^{res}$.
