# Supplementary material for: Prominent and Persistent Extraneural Infection in Human PrP Transgenic Mice Infected with Variant CJD
Source: PLoS One. 2008 Jan 9;3(1):e1419. doi: 10.1371/journal.pone.0001419 (PMC2171367; doi:10.1371/journal.pone.0001419)
Supplement: Table S1 — Differential distribution of PrPres in the brains of tg650 mice infected with early or late brain passage (0.05 MB DOC) [file pone.0001419.s006.doc]

**Table S1.** Differential distribution of PrP^res^ in the brains of tg650 mice infected with *early* or *late* brain passage.

|  | sCJD | vCJD no.4 | | vCJD |
| --- | --- | --- | --- | --- |
| Brain Regions | no.1-4 | *Early* | *Late* | no.1-3 |
| Cerebral cortex | - | - | ++ | ++ |
| Septum | + | + | ++ | ++ |
| Striatum | -/+ | -/+ | + | + |
| Ventral pallidum | - | - | + | + |
| Corpus callosum | - | - | +++ | +++ |
| Molecular layer of the hippocampus | - | - | + | + |
| Optic and lateral optic tract | - | - | ++ | ++ |
| Thalamus |  |  |  |  |
| Lateral posterior nuclei | ++ | ++ | + | + |
| Laterodorsal nuclei | ++ | ++ | + | + |
| Geniculate nuclei | ++ | ++ | + | + |
| Posterior nuclei | - | - | ++ | ++ |
| Ventral posteromedial nuclei | - | - | ++ | ++ |
| Central medial nuclei | ++ | ++ | + | + |
| Lateral hypothalamus | -/+ | -/+ | + | + |
| Brain stem (raphe + pontine reticular nuclei) | -/+ | -/+ | ++ | ++ |
| Inferior colliculus (external cortex) | -/+ | -/+ | -/+ | -/+ |
| Cerebellum | - | - | + | + |

-: no staining; -/+: inconsistent staining; +: moderate; ++: pronounced; +++, intense staining.
