# Supplementary material for: Prominent and Persistent Extraneural Infection in Human PrP Transgenic Mice Infected with Variant CJD
Source: PLoS One. 2008 Jan 9;3(1):e1419. doi: 10.1371/journal.pone.0001419 (PMC2171367; doi:10.1371/journal.pone.0001419)
Supplement: Text S1 — Development of the human PrP transgenic mice (tg650 line) (0.03 MB DOC) [file pone.0001419.s007.doc]

**Text S1: Development of the human PrP transgenic mice (tg650 line)**

In order to avoid as much as possible effects that could be due to a PrP ectopic expression, we have constructed transgenic mice using a human PAC (P1-derived artificial chromosome) containing the PrP gene and its flanking elements. The RCP PAC library has been screened by PCR with primers derived from the human PrP sequence. One of the isolated PAC had a 110 kb-long insert encompassing the whole human PrP gene as well as flanking genes (such as doppel). The PrP gene ORF was sequenced and shown to contain the Met ATG codon at position 129.

The insert of this PAC was purified and microinjected into murine *Prnp*-knockout oocytes [1]. Five transgenic founders were obtained. FISH experiments revealed that two lines have integrated PAC copies in two locations and these lines were discarded. The three remaining lines had respectively integrated 1, 3 and 5 copies of the PAC. Using a RT-PCR assay, the expression of the human PrP transgene was shown to be a linear function of the number of copies of the PAC that have been integrated in the murine genome. The 5 copies line (tg650 line), which expresses the highest amount of human PrP^C^ (6-fold for the homozygous mice as compared with human brain tissue), has been further explored. These mice, even when homozygous at the transgene locus, do not exhibit any abnormal phenotype or neurological signs and have a normal lifespan around 2 years. PrP^C^ expression levels in the spleen of these mice were 20-fold lower compared to the brain, similar to that in conventional mice (Figure S1).

The production of the tg650 line and of other human PrP transgenic mouse lines was achieved by a group of laboratories involved into a network supported by a grant from the French Ministry of Research, including: INSERM, Faculté de Médecine de la Timone, Marseille (M. Fontés), and Institut Pasteur de Lille (J.Y. Cesbron); INRA, Unités de Génétique Biochimique et Cytogénétique (J.L. Vilotte) and Biologie du Développement et Reproduction (L.M. Houdebine), Jouy-en-Josas.

**Reference:**

1. Bueler H, Fischer M, Lang Y, Bluethmann H, Lipp HP, et al. (1992) Normal development and behaviour of mice lacking the neuronal cell-surface PrP protein. Nature 356: 577-582.
